# Data from a survey of *Clostridium perfringens* and *Clostridium difficile* shedding by dogs and cats in the Madrid region (Spain), including phenotypic and genetic characteristics of recovered isolates

**DOI:** 10.1016/j.dib.2017.07.029

**Published:** 2017-07-15

**Authors:** Sergio Álvarez-Pérez, José L. Blanco, Celine Harmanus, Ed J. Kuijper, Marta E. García

**Affiliations:** a Department of Animal Health, Faculty of Veterinary, Universidad Complutense de Madrid, Madrid, Spain; b Department of Medical Microbiology, Center of Infectious Diseases, Leiden University Medical Center, Leiden, The Netherlands

**Keywords:** Antimicrobial resistance, Cat, *Clostridium difficile*, *Clostridium perfringens*, Dog, Genetic diversity

## Abstract

This article contains information related to a recent survey of the prevalence of fecal shedding of *Clostridium perfringens* and *C. difficile* by dogs and cats attended in veterinary clinics located in the Madrid region (Spain). Specifically, we provide detailed information about the clinics that participated in the survey, the demographic and clinic characteristics of recruited animals and the genetic and phenotypic characteristics (including antimicrobial susceptibility data), of recovered bacterial isolates.

**Specifications Table**

**Table:** 

Subject area	*Biology*
More specific subject area	*Veterinary microbiology, anaerobes, Clostridium perfringens, Clostridium difficile*
Type of data	*Tables, figures and text*
How data was acquired	*Analysis of clinical data and characteristics of bacterial isolates*
Data format	*Filtered and analyzed*
Experimental factors	*Dogs and cats attended in veterinary clinics, and the Clostridium perfringens and C. difficile isolates obtained from their feces*
Experimental features	*Analysis of general data about participating clinics, and the demographic and clinical features of recruited animals; genetic and phenotypic profiling of isolates*
Data source location	*Universidad Complutense de Madrid, Madrid, Spain*
Data accessibility	*Data is provided with this article*

**Value of the data**

• First detailed analysis of the prevalence of *Clostridium perfringens* and *Clostridium difficile* shedding by small animals (dogs and cats) in the Madrid region (Spain).
• Detailed phenotypic and genetic data of recovered isolates is provided, which may be useful for comparison in future epidemiological surveys.
• Given the recent emergence of antibiotic-resistant strains of *C. difficle*, information on the antimicrobial susceptibility profiles of the isolates obtained in this survey may be particularly valuable.

## 1 Data

The data shown in Section 1.1 of this article provide detailed information on the veterinary clinics that participated in a recent survey of the prevalence of fecal shedding of *Clostridium perfringens* and *C. difficile* by dogs and cats which was carried out in the Madrid region (Spain) [1]. Furthermore, the demographic and clinical features of recruited animals are detailed in Section 1.2, and Section 1.3 provides extensive data on the genetic and phenotypic characteristics of recovered bacterial isolates.

### 1.1 General data about participating clinics

An overview of the 17 veterinary clinics that participated in the study (hereafter referred to as clinics A to Q) is provided in Table 1. Two clinics (L and P) did not return a questionnaire of general data about their centre (see Section 2) and in two other cases (clinics H and K) the returned questionnaire was incomplete. Participating clinics were scattered within the Madrid region (14 were located in the capital city, two in other municipalities within the metropolitan area and one in a rural location) and varied widely in their year of opening (from 1981 to 2014), number of cases attended per week (x±S.D.=37.6±18.7 and 16.5±11 for dogs and cats, respectively), number of fecal cultures requested per week (1.6±2.4 and 0.9±1.8), and other parameters (Table 1). These clinics also differed in the antibiotics used for the treatment of diarrhea, but 12 of them (80% for which pharmacological data were available) reported the use of metronidazole for the treatment of these conditions. Only three clinics (20%; F, J and O) acknowledged frequent request of microbiological culturing for anaerobes, and five clinics (33.3%; D, F, H, N and O) reported occasional suspicion of *C. difficile* and/or *C. perfringens* involvement in severe cases of diarrhea.

**Table 1:** Overview of the characteristics of the veterinary clinics that participated in the study.^a^

**Clinic**	**Opening year**	**No. cases per week**^b^	**No. diarrea cases per week**^b^	**No. fecal samples per week**^b^	**No. fecal cultures per week**^b^
A	2014	61 (55 D, 6 C)	2.5 (2 D, 0.5 C)	1 (1 D)	0.5 (0.5 D)
B	1994	82 (50 D, 32 C)	2.5 (2 D, 0.5 C)	3.5 (3 D, 0.5 C)	0
C	2009	11 (10 D, 1 C)	5.5 (5 D, 0.5 C)	1 (1 D)	0
D	2014	40 (22 D, 18 C)	8 (7 D, 1 C)	7 (5 D, 2 C)	0
E	1993	40 (20 D, 20 C)	2 (2 D)	4 (2 D, 2 C)	4 (2 D, 2 C)
F	1981	100 (70 D, 30 C)	14 (10 D, 4 C)	2.3 (2 D, 0.3 C)	0.31 (0.3 D, 0.01 C)
G	1984	27.5 (16 D, 11.5 C)	11.5 (8 D, 3.5 C)	1.5 (1 D, 0.5 C)	1.5 (1 D, 0.5 C)
H	2002	55 (35 D, 20 C)	5 (4 D, 1 C)	NA	NA
I	1992	70 (60 D, 10 C)	8 (7 D, 1 C)	1.5 (1 D, 0.5 C)	0
J	2004	90 (48 D, 42 C)	12 (8 D, 4 C)	16 (10 D, 6 C)	14 (8 D, 6 C)
K	1985	NA	NA	10 (6 D, 4 C)	10 (6 D, 4 C)
L	NA	NA	NA	NA	NA
M	2001	47.5 (35 D, 12.5 C)	4.5 (4 D, 0.5 C)	4.5 (4 D, 0.5 C)	3 (2.5 D, 0.5 C)
N	1999	35 (25 D, 10 C)	4 (3 D, 1 C)	4 (3 D, 1 C)	1 (1 D)
O	2010	26 (20 D, 6 C)	3 (2.5 D, 0.5 C)	0.5 (0.5 D)	0.5 (0.5 D)
P	NA	NA	NA	NA	NA
Q	2005	72 (60 D, 12 C)	20 (20 D)	2 (2 D)	0
Total^c^		54.1±25.3 (37.6±18.7 D, 16.5±11 C)	7.3±5.1 (6±4.6 D, 1.3±1.4 C)	4.2±4.1 (3±2.5 D, 1.2±1.7 C)	2.5±4.1 (1.6±2.4 D, 0.9±1.8 C)

a See also Fig. 1.

b D, dogs; C, cats. All figures refer to the last 12-month period.

c x±S.D.

### 1.2 Demographic and clinical features of recruited animals

**Fig. 1:**
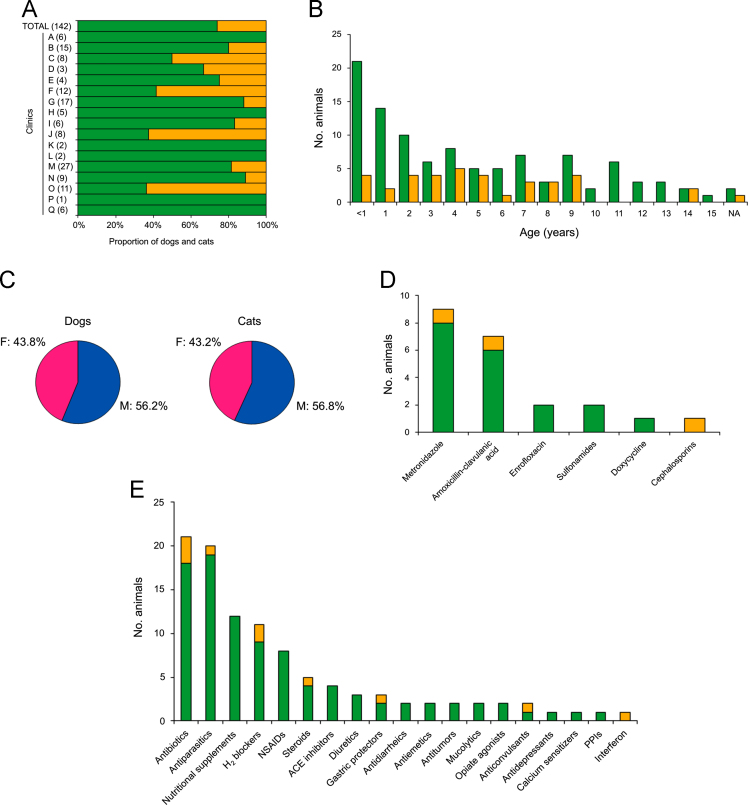
Characteristics of the animal populations recruited for this study. A) Bar plot showing the distribution of recruited animals per species: dog, green bars; cats, orange bars. For each participating clinic (A to Q), the overall number of animals is indicated between parentheses. B) Bar plot of the overall age distribution of dogs (*n*=105; green bars) and cats (*n*=37; orange bars) recruited for the study. C) Pie charts of the overall sex distribution of dogs (n=105) and cats (n=37) included in the study. Blue and pink sectors represent male (M) and female (F) subpopulations, respectively. D) Bar plot of the antimicrobial treatments administered to recruited dogs and cats (green and orange bars, respectively) ≤30 days before sampling. E) Bar plot of recent pharmacological treatments administered to recruited dogs and cats (green and orange bars, respectively) ≤30 days before sampling. Abbreviations: ACE, angiotensin-converting-enzyme; NSAIDs, nonsteroidal anti-inflammatory drugs; PPIs, proton pump inhibitors. In panels D and E, pharmacological treatment data were not available for a total of 48 animals (32 dogs and 16 cats).

**Fig. 2:**
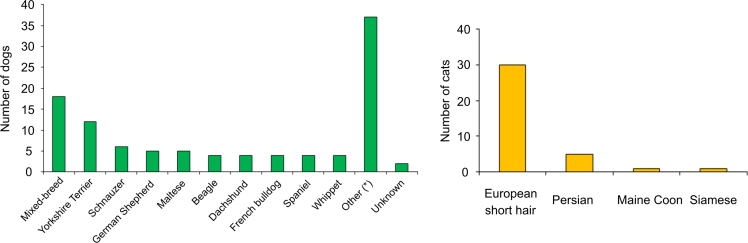
Bar plots showing the breed distribution of recruited dogs (*n*=105, left panel) and cats (*n*=37, right panel). *Other dog breeds (*n*): Boxer (3), Setter (3), Shih Tzu (3), American Staffordshire Terrier (2), Belgian Shepherd (2), Border Collie (2), Chinese Shar-Pei (2), Cocker Spaniel (2), Golden Retriever (2), Labrador Retriever (2), Pug (2), Shiba Inu (2), Andalusian Hound (1), Chihuahua (1), Dalmatian (1), Galician Palleiro (1), Hound (1), Mastiff (1), Miniature Pinscher (1), Pit bull (1), Poodle (1), Water dog (1).

The demographic characteristics of recruited animals are summarized in Table 2 and Fig. 1, Fig. 2. A total of 142 animals, including 105 dogs and 37 cats (73.9% and 26.1% of total, respectively; Fig. 1A) of diverse breeds (Fig. 2), were recruited for the study. The male/female ratio of animals varied widely among clinics, with the overall values for dogs and cats being similar (56.2%, 43.8% and 56.8%, 43.2%, respectively; Fig. 1C). The age distribution of sampled animals also showed ample variation among clinics, but the overall values were similar for the dog and cat subpopulations: 20%, 45.7%, 32.4% of dogs and 10.8%, 54.1%, 32.4% of cats had <1 year, 1–6 years and ≥7 years, respectively (Table 2).

**Table 2:** Overview of the animals that were recruited for this study^a^.

**Clinic**	***N***^b^	**Dogs**								**Cats**							
		***n***	**Sex ratio**^c^	**Age distribution**			**Days since last episode of diarrhea**			***n***	**Sex ratio**^c^	**Age distribution**			**Days since last episode of diarrhea**		
				**<1 yr**	**1–6 yr**	**≥7 yr**	**0**	**≤30**	**>30**			**<1 yr**	**1–6 yr**	**≥7 yr**	**0**	**≤30**	**>30**
A	6	6	2:1	33.3%	66.7%	0%	0%	66.7%	33.3%	0	–	–	–	–	–	–	–
B	15	12	1:5	25%	25%	50%	25%	0%	75%	3	2:1	0%	33.3%	66.7%	0%	0%	100%
C	8	4	3:1	50%	50%	0%	0%	25%	75%	4	1:1	0%	25%	75%	50%	0%	50%
D	3	2	1:1	0%	100%	0%	0%	0%	100%	1	0:1	0%	0%	100%	0%	0%	100%
E	4	3	2:1	0%	66.7%	33.3%	0%	66.7%	33.3%	1	0:1	0%	0%	100%	100%	0%	0%
F	12	5	2:3	0%	20%	80%	0%	40%	60%	7	4:3	14.3%	85.7%	0%	0%	0%	100%
G	17	15	11:4	20%	40%	40%	13.3%	26.7%	60%	2	0:1	0%	100%	0%	0%	0%	100%
H	5	5	4:1	100%	0%	0%	20%	20%	60%	0	–	–	–	–	–	–	–
I	6	5	4:1	20%	60%	20%	40%	20%	40%	1	1:0	0%	0%	100%	0%	100%	0%
J*	8	3	2:1	0%	66.7%	0%	33.3%	66.7%	0%	5	3:2	20%	60%	0%	40%	60%	0%
K	2	2	1:0	0%	50%	50%	0%	0%	100%	0	–	–	–	–	–	–	–
L*	2	2	0:1	0%	0%	50%	50%	0%	50%	0	–	–	–	–	–	–	–
M	27	22	4:7	4.5%	59.1%	36.4%	0%	13.6%	86.4%	5	3:2	20%	60%	20%	0%	0%	100%
N*	9	8	7:1	12.5%	37.5%	50%	12.5%	0%	37.5%	1	1:0	0%	0%	100%	0%	0%	100%
O	11	4	1:1	25%	75%	0%	0%	25%	75%	7	5:2	14.3%	57.1%	28.6%	0%	0%	100%
P	1	1	0:1	100%	0%	0%	100%	0%	0%	0	–	–	–	–	–	–	–
Q	6	6	5:1	16.7%	50%	33.3%	33.3%	33.3%	33.3%	0	–	–	–	–	–	–	–
Total*	142	105	59:46	20%	45.7%	32.4%	13.3%	21.9%	61%	37	21:16	10.8%	54.1%	32.4%	13.5%	10.8%	75.7%

a See also Fig. 1. Asterisks indicate that there were some recruited animals with missing data and thus the sum of percentages can be <100%.

b Total number of animals that were recruited (i.e. dogs and cats).

c Number of males: number of females.

The overall proportion of dogs and cats with diarrhea on the sampling date were very similar (13.3% and 13.5%, respectively), and in both cases most animals had not suffered any episode of diarrhea within the preceding 30 days (61% and 75.7%, respectively) (Table 2). Only 24.7% (18/73) of dogs and 14.3% (3/21) of cats for which medication data was available were under antibiotic treatment on the sampling date or within the previous 30 days, with metronidazole and amoxicillin ranking first and second, respectively (Fig. 1D). Other pharmacological treatments of sampled animals are shown in Fig. 1E.

The demographic data and clinical features of animals yielding positive fecal cultures for *C. perfringens* and/or *C. difficile* are detailed in Table 3 (see also Álvarez-Pérez et al. [1]).

**Table 3:** Signalment and clinical data of animals yielding positive fecal cultures for *Clostridium perfringens* and/or *C. difficile*, and characteristics of recovered isolates.

**Clinic**	**Animal**^a^	**Species, breed**^b^	**Age (yr.)**^b^	**Sex**	**Diagnosis**	**Other medical conditions of relevance**	**Diarrhoea**^c^	**Antibiotic treatment(s)**^d^	**Genotype (antimicrobial resistances) of*****C. perfringens*****isolates**^e^	**Genotype (antimicrobial resistances) of*****C. difficile*****isolates**^f^
A	A/02	Dog, Mixed-breed	5	F	Routine analysis	None	No	None	p063, p064, p065	–
B	B/02	Dog, Whippet	13	F	Routine analysis	Lymphoma	No	None	p067	–
	B/06	Dog, Whippet	7	F	Routine analysis	None	No	None	p027, p028 [2 isolates]	–
	B/07	Dog, Schnauzer	9	F	Routine analysis	None	No	None	p011, p012, p023	–
	B/08	Dog, Schnauzer	9	F	Routine analysis	None	No	None	p002, p047, p051	RT154/d05 (PEN), RT154/d06 (PEN), RT154/d07 (PEN)
	B/11	Dog, Mixed Setter	<1	F	Recent history of *Isospora* infection	None	Yes	Metronidazole	p035, p036 [2 isolates]	–
	B/12	Dog, Mixed Miniature Pinscher	6	F	Routine analysis	None	No	None	p037	–
	B/13	Cat, European shorthair	9	F	Routine analysis	None	No	None	p003 (MTZ), p006, p031	–
	B/14	Cat, European shorthair	1	M	Routine analysis	None	No	None	p069, p072	–
D	D/02	Dog, Hound	1	F	Routine analysis	None	No	None	p087 (MTZ), p088 (LZD), p095	–
E	E/03	Dog, Yorkshire Terrier	11	M	Gastroenteritis with vomiting	Heart failure	Yes (5 d)	Amoxicillin-clavulanic acid	NA	RT?/d13 (CLI/ERY/PEN) [2 isolates], RT?/d14 (CLI/ERY/PEN)
F	F/09	Cat, Persian	6	M	Routine analysis	None	No	None	p004, p005	–
G	G/01	Dog, Labrador	12	M	Routine analysis	None	Yes (7 d)	Doxycycline	p106	–
					Follow-up analysis	None	No	None	p041, p092, p097	–
	G/05	Dog, Boxer	<1	M	Routine analysis	Food allergy	No	None	p030 (MTZ), p050, p096	–
	G/06	Cat, Persian	2	F	Routine analysis	None	No	None	p026	–
H	H/03	Dog, Mastiff	<1	F	Routine analysis	None	No	None	p078 (PEN), p102 (IPM, PEN), p103	–
	H/05	Dog, Pit bull	<1	M	Routine analysis	None	No	None	p008, p034, p079 (IPM, LZD, PEN)	–
I	I/02	Cat, European shorthair	14	M	Digestive disease	None	Yes (3 d)	None	p054, p055	–
	I/04	Dog, Mixed-breed	1	H	Digestive disease	None	Yes (0 d)	None	p082, p099	–
					Follow-up analysis	None	No	None	p052 (LZD)	–
J	J/01	Dog, Schnauzer	?	F	Routine analysis	Food allergy	Yes (10 d)	None	p053, p058 (ERY, LVX)	–
	J/03	Dog, Golden Retriever	4	M	Acute enteritis	None	Yes (0 d)	None	p013, p017, p018	–
	J/07	Dog,?	1	M	Routine analysis	None	Yes (8 d)	None	p101	–
K	K/02	Dog, Poodle	10	M	Routine analysis	Heart murmur	No	None	p089	–
M	M/04	Dog, Giant Schnauzer	11	F	Routine analysis	None	Yes (30 d)	None	p090[2 isolates], p091	–
					Follow-up analysis	None	Yes (5 d)	None	p043 (TET), p076 (TET), p077	–
	M/06	Dog, Brittany	6	F	Routine analysis	Obesity	No	None	p060, p061, p066	–
					Follow-up analysis	Obesity	No	None	p068, p070 (TET), p104	–
	M/07	Dog, Brittany	3	F	Entetitis	Recent *Toxocara canis* infection, obesity	Yes (25 d)	None	p081	–
					Follow-up analysis	Obesity	No	None	p033, p042 (TET), p056	–
	M/08	Dog, Mixed Hound	3	F	Routine analysis	None	No	None	p040, p062, p080	–
	M/13	Cat, European shorthair	4	F	Routine analysis	None	No	None	p025, p029, p071	–
	M/14	Dog, Mixed-breed	8	F	Routine analysis	Recent removal of mammary tumor	No	Amoxicillin	p016, p020, p021	RT106/d01 (PEN), RT106/d03 (PEN), RT106/d15 (PEN)
					Follow-up analysis	None	No	None	p014[2 isolates], p015	–
	M/23	Dog, Yorkshire Terrier	12	F	Routine analysis	Frequent pseudo-pregnancies, benign mammary tumors	No	None	p032	–
	M/25	Dog, German Shepherd	5	F	Routine analysis	Obesity	No	None	p001 (TET), p057	–
					Follow-up analysis	Obesity	No	None	p083 (TET), p084 (LZD, TET), p085 (LZD. TET)	–
	M/26	Dog, Mixed-breed	2	M	Routine analysis	Seizure disorder	No	None	p038, p039	–
					Follow-up analysis	Seizure disorder	No	None	p086 (MTZ), p093, p100	–
N	N/01	Dog, Mixed Shih Tzu	1	M	Routine analysis	None	No	None	p044, p045, p046	–
	N/12&14^g^	Dog, Shih Tzu	12	M	Routine analysis	None	Yes (0 d)	None	p019, p024	–
	N/19&20^g^	Dog, French Bulldog	7	M	Routine analysis	None	No	None	–	RT009-like/d04, RT009-like/d08 (PEN), RT009-like/d09 (PEN), RT009-like/d10 (PEN), RT009-like/d11 (PEN), RT009-like/d12
O	O/04	Cat, European shorthair	4	M	Routine analysis	None	No	None	p094	–
	O/11	Dog, Border Collie	2	M	Routine analysis	None	Yes (7 d)	None	p007, p009, p010	–
Q	Q/03	Dog, Maltese	7	M	Routine analysis	Frequent gastrointestinal problems, heart disease	No	None	p073 (MTZ), p074, p075 (LZD)	RT106/d01 (PEN) [2 isolates], RT106/d02 (PEN)
	Q/04	Dog, Mixed Labrador	2	M	Allergic colitis	Frequent gastrointestinal problems	Yes (0 d)	Sulfadiazine/trimethoprim	p059	–
					Follow-up analysis	None	No	None	p022, p098	–
	Q/05	Dog, Dachshund	14	M	Routine analysis	None	No	None	p048, p049	–

a Recruited animals were designated by a capital letter (corresponding to the clinic of origin) followed by a virgule and consecutive numbers.

b ?: Missing data.

c No: no diarrhea episodes were reported within the previous 30 days. For positive responses, the number of days since the last episode of diarrhea is indicated between parentheses.

d Antibiotic treatment(s) administered within the previous 30 days. For an overview of other pharmacological treatments see Fig. 1E.

e AFLP genotypes were arbitrarily designated by a lower case ‘p’ followed by a number (see Table 4). *In vitro* resistance to benzylpenicillin (PEN), erythromycin (ERY), imipenem (IPM), levofloxacin (LVX), linezolid (LZD), metronidazole (MTZ) and/or tetracycline (TET) is indicated between parentheses. The number of isolates belonging to each strain type (when different to one) is shown between square brackets. Dashes mean that *C. perfringens* was not isolated from the corresponding animal. NA: not analyzed (a single fecal swab was available for some animals, and this was used for *C. difficile* testing).

f In this case, ribotype (RT) and AFLP fingerprinting information is included. AFLP genotypes were arbitrarily designated by a lower case ‘d’ followed by a number (see Table 5). *In vitro* resistance to benzylpenicillin (PEN), clindamycin (CLI) and/or erythromycin (ERY) is indicated between parentheses. Additionally, all *C. difficile* isolates displayed resistance to levofloxacin and imipenem. The number of isolates belonging to each strain type (when different to one) is shown between square brackets. RT?: unknown ribotype. Dashes mean that *C. difficile* was not isolated from the corresponding animal.

g These animals were sampled twice during the study period.

### 1.3 Genetic and phenotypic characteristic of *C. perfringens* and *C. difficile* isolates

Table 3 includes an overview of the genetic and phenotypic characteristics of the bacterial isolates obtained from recruited animals. Additionally, the toxinotypes, PCR ribotypes (only for *C. difficile* isolates), amplified fragment length polymorphism (AFLP) genotypes and antimicrobial susceptibility profiles of *C. perfringens* and *C. difficile* isolates are detailed in Table 4, Table 5, respectively.

**Table 4:** Characteristics of the *Clostridium perfringens* isolates obtained in the study.

**Isolate**^a^	**Toxinotype**^b^	**AFLP genotype**^c^	**Antibiotic susceptibility (MIC, µg/ml)**^d^												
			**AMC**	**CLI**	**ERY**	**IPM**	**LVX**	**LZD**	**MTZ**	**PEN**	**RIF**	**TEC**	**TET**	**TGC**	**VAN**
A/02P1	A (*cpe*, cpb2*)	p063	0.125	0.25	2	0.064	0.5	4	16	0.5	0.008	0.064	8	1	1
A/02P2	A (cpb2*)	p064	0.032	0.032	0.064	0.064	0.25	0.125	8	0.125	0.004	0.032	4	0.125	0.5
A/02P3	A (*cpe*)	p065	0.032	0.032	0.032	0.064	0.25	0.125	8	0.064	0.002	0.032	0.125	0.064	0.5
B/02P1	A	p067	0.032	0.064	2	0.064	0.25	4	16	0.064	0.008	<0.016	0.125	0.064	0.5
B/06P1	A (cpb2*)	p027	0.064	0.064	2	0.064	0.25	4	16	0.032	0.004	<0.016	4	0.25	0.5
B/06P2	A (cpb2*)	p028	0.032	0.125	2	0.5	0.25	4	16	0.125	0.004	0.032	4	0.5	0.5
B/06P3	A (cpb2*)	p028	<0.016	0.5	2	0.5	0.25	4	16	0.064	0.008	0.032	4	0.5	0.5
B/07P1	A (*cpe*)	p011	0.032	0.125	1	0.032	0.25	4	16	0.032	0.004	<0.016	2	0.125	0.25
B/07P2	A (*cpe*)	p012	0.032	0.125	2	0.064	0.25	4	16	0.064	0.008	0.032	4	0.125	0.25
B/07P3	A (*cpe*)	p023	<0.016	0.125	2	0.064	0.25	2	16	0.125	0.008	0.032	4	0.125	0.25
B/08P1	A (cpb2*)	p047	0.032	0.064	2	0.125	0.25	4	16	0.064	0.004	<0.016	4	0.125	0.5
B/08P2	A (cpb2*)	p002	0.032	0.25	2	0.064	0.25	2	16	0.064	0.008	0.032	2	0.5	0.5
B/08P3	A (cpb2*)	p051	0.016	0.125	2	0.125	0.25	4	16	0.064	0.008	0.032	4	0.25	0.5
B/11P1	A (cpb2*)	p035	0.125	0.016	1	0.064	0.25	1	16	0.125	0.004	<0.016	4	0.125	0.5
B/11P2	A (cpb2*)	p036	0.016	0.25	2	0.064	0.25	2	8	0.125	0.004	0.016	4	0.5	0.5
B/11P3	A (cpb2*)	p036	0.032	0.25	2	0.008	0.5	2	16	0.125	0.004	0.032	4	0.5	0.5
B/12P1	A	p037	0.032	0.016	0.125	0.064	0.25	1	8	0.032	0.004	<0.016	1	0.125	0.5
B/13P1	A (cpb2*)	p003	0.064	0.064	2	0.032	0.5	4	32 (R)	0.064	0.008	0.032	2	0.064	0.5
B/13P2	A	p006	0.032	0.064	2	0.25	0.5	4	16	0.125	0.004	0.064	8	0.125	0.5
B/13P3	A	p031	0.064	0.25	2	0.064	0.25	4	16	0.125	0.008	<0.016	8	0.125	0.5
B/14P1	A	p069	0.064	0.125	2	0.064	0.5	2	16	0.25	0.008	0.032	4	0.25	0.5
B/14P2	A	p072	0.032	2	2	0.032	0.25	<0.016	8	0.064	0.004	<0.016	4	0.25	0.5
D/02P1	E (*cpe*)	p087	0.064	<0.016	1	0.5	0.25	2	32 (R)	0.064	0.002	0.032	4	0.064	0.5
D/02P2	E (*cpe*)	p088	0.064	0.064	4	0.125	0.25	8 (R)	8	0.125	0.004	0.032	4	0.25	1
D/02P3	E (*cpe*)	p095	0.032	0.032	1	0.064	0.5	1	16	0.125	0.004	0.032	2	0.25	0.5
F/09P2	A	p004	0.125	0.064	0.5	0.25	0.25	0.5	8	0.25	0.004	0.032	0.125	0.064	0.5
F/09P3	A	p005	0.064	0.064	2	1	0.25	4	8	0.25	0.008	0.064	4	0.064	1
G/01P1	A	p105	0.064	0.064	0.064	0.064	0.5	1	16	0.064	0.004	<0.016	1	0.125	0.5
G2/01P1†	A (cpb2*)	p092	0.064	0.064	4	0.125	0.25	4	8	0.125	0.008	0.064	4	0.25	0.5
G2/01P2†	A (cpb2*)	p097	0.064	<0.016	0.064	0.125	0.25	4	16	0.125	0.008	0.016	4	0.064	0.5
G2/01P3†	A (cpb2*)	p041	0.032	0.125	2	0.064	0.25	4	8	0.125	0.004	0.016	8	0.25	0.5
G/05P1	A (cpb2*)	p030	0.125	0.064	2	0.064	0.25	4	32 (R)	0.125	0.008	0.032	8	0.125	0.5
G/05P2	A (cpb2*)	p050	0.064	0.25	1	0.064	0.25	2	8	0.5	0.004	<0.016	4	0.25	0.5
G/05P3	A	p096	<0.016	0.125	1	0.032	0.5	4	4	0.125	0.004	<0.016	4	0.125	0.5
G/06P1	A	p026	0.125	0.125	2	0.064	0.5	4	16	0.125	0.008	0.032	8	0.25	1
H/03P1	A	p102	8	0.125	2	≥32 (R)	0.25	4	16	8 (R)	0.004	0.032	8	0.125	0.5
H/03P2	A	p078	4	0.032	2	1	0.25	4	16	8 (R)	0.004	0.032	4	0.125	0.5
H/03P3	A	p103	0.032	0.064	1	0.125	2	2	8	0.064	0.004	0.064	8	0.5	0.5
H/05P1	A	p079	8	0.125	2	≥32 (R)	0.25	8 (R)	16	8 (R)	0.004	0.032	8	0.5	0.5
H/05P2	A	p008	0.032	0.125	1	0.064	0.25	1	4	0.064	0.002	0.016	4	0.125	0.5
H/05P3	A	p034	<0.016	0.064	2	0.125	0.25	4	16	0.032	0.008	0.032	4	0.125	0.5
I/02P1	A	p054	0.064	0.064	2	0.125	0.25	2	16	0.125	0.004	0.016	4	0.125	0.5
I/02P2	A	p055	0.032	<0.016	4	0.064	0.5	0.25	16	0.064	<0.002	<0.016	4	0.064	0.5
I/04P1	A (*cpe*)	P099	0.125	0.032	0.25	0.125	0.25	1	16	0.125	0.004	0.032	0.125	0.032	0.5
I/04P2	A	p082	0.032	0.25	2	0.032	0.25	0.5	16	0.125	<0.002	<0.016	4	0.125	0.5
I2/04P1†	A (*cpe*)	p052	0.032	0.125	2	0.064	0.25	8 (R)	16	0.125	0.008	0.032	4	0.25	0.5
J/01P1	A (cpb2*)	p053	0.064	0.125	2	0.064	0.5	4	16	0.125	0.008	0.032	8	0.5	1
J/01P2	A (cpb2*)	p058	0.064	0.25	32 (R)	0.125	≥32 (R)	2	16	0.125	0.008	0.032	4	0.125	0.5
J/03P1	A	p018	0.064	0.032	2	0.064	0.5	4	16	0.064	0.004	<0.016	0.125	0.064	0.5
J/03P2	A (cpb2*)	p013	0.125	0.032	0.125	0.064	0.25	4	16	0.064	<0.002	2	1	0.032	2
J/03P3	A (cpb2*)	p017	0.016	0.032	0.125	0.032	0.25	2	16	0.064	0.004	<0.016	2	0.032	0.5
J/07P1	A (*cpb2*)	p101	0.016	<0.016	0.125	0.064	0.25	1	16	0.032	0.008	0.032	0.125	0.032	0.5
K/02P1	A	p089	0.032	0.125	2	0.5	0.25	4	16	0.125	0.004	0.032	4	0.125	0.5
M/04P1	A (*cpe*)	p090	0.064	0.125	2	0.064	0.25	4	16	0.125	0.008	0.064	8	0.5	0.5
M/04P2	A (*cpe*)	p090	0.032	0.125	2	1	0.5	4	16	0.064	0.004	0.064	4	0.5	0.5
M/04P3	A (*cpe*)	p091	0.032	<0.016	<0.016	0.5	0.25	4	16	0.064	0.016	0.064	4	0.125	0.5
M2/04P1†	A (cpb2*)	p043	0.032	0.032	2	0.25	0.25	4	16	0.032	0.004	0.016	16 (R)	1	0.5
M2/04P2†	A (cpb2*)	p076	0.032	0.064	2	0.5	0.25	4	8	0.064	0.008	0.016	16 (R)	0.5	0.5
M2/04P3†	A (cpb2*)	p077	0.032	0.032	0.064	0.064	0.5	2	8	0.064	0.004	0.016	4	0.25	0.5
M/06P1	A (cpb2*)	p066	0.125	0.064	2	0.064	0.25	4	16	0.5	0.008	0.064	8	2	1
M/06P2	A (cpb2*)	p060	0.032	0.064	1	0.064	0.25	2	16	0.125	0.004	0.032	4	0.25	0.5
M/06P3	A (*cpe*, cpb2*)	p061	0.032	0.032	0.5	0.125	0.25	4	16	0.125	0.004	0.032	4	0.125	0.5
M2/06P1†	A	p068	0.064	0.064	2	0.5	0.5	4	16	0.25	0.008	0.032	8	0.125	0.5
M2/06P2†	A	p070	0.016	0.125	2	0.032	0.25	4	16	0.125	0.004	0.032	16 (R)	0.5	0.5
M2/06P3†	A	p104	0.064	0.032	0.25	0.125	0.25	4	8	0.125	0.008	0.016	4	0.5	0.5
M/07P1	A	p081	0.25	0.032	2	0.125	0.25	4	8	1	0.008	0.032	4	0.5	0.5
M2/07P1†	A (cpb2*)	p042	<0.016	0.032	2	0.064	0.25	4	16	0.125	0.008	0.016	16 (R)	1	0.5
M2/07P2†	A	p056	0.032	0.064	4	0.125	0.25	4	8	0.125	0.004	0.032	4	0.25	0.5
M2/07P3†	A	p033	0.032	0.064	1	0.125	0.5	4	8	0.25	0.008	0.032	8	0.25	0.5
M/08P1	A (cpb2*)	p062	0.064	0.5	2	0.064	0.25	4	16	0.25	<0.002	0.032	8	2	0.5
M/08P2	A	p040	0.032	0.064	0.25	0.064	0.25	4	16	0.125	0.008	0.016	1	0.064	0.5
M/08P3	A	p080	0.032	<0.016	0.032	0.064	0.5	1	8	0.064	<0.002	0.032	4	0.125	0.5
M/13P1	A (cpb2*)	p029	0.125	0.25	2	0.064	0.25	4	16	0.064	0.004	0.032	8	4	1
M/13P2	A (cpb2*)	p025	0.032	0.032	0.032	0.032	0.25	0.125	8	0.064	<0.002	0.032	4	0.032	0.5
M/13P3	A (*cpe*)	p071	0.032	1	2	0.125	0.5	2	8	0.064	0.002	0.032	4	0.125	0.5
M/14P1	A (*cpe*)	p016	0.016	0.064	2	0.064	0.25	4	8	0.064	0.004	0.016	0.125	0.064	0.5
M/14P2	A (*cpe*)	p020	0.125	0.5	2	0.064	0.25	2	4	0.125	0.004	<0.016	2	0.032	0.5
M/14P3	A (*cpe*)	p021	0.032	0.125	2	0.032	0.25	2	8	0.064	0.008	0.016	4	0.125	0.5
M2/14P1†	A (cpb2*)	p014	<0.016	0.032	2	0.125	0.25	1	8	0.064	<0.002	0.016	4	0.125	0.5
M2/14P2†	A (cpb2*)	p015	0.064	0.032	0.032	0.064	0.25	2	16	0.064	0.004	0.016	4	0.5	0.5
M2/14P3†	A (cpb2*)	p014	0.064	0.032	2	0.125	0.25	2	16	0.064	0.004	0.016	4	0.5	0.5
M/23P1	A (*cpb2*)	p032	0.064	0.25	2	0.016	0.25	2	16	0.25	0.008	0.032	4	0.125	1
M/25P1	A	p001	0.125	2	4	0.064	0.5	4	16	0.25	0.008	0.125	16 (R)	2	1
M/25P2	A	p057	0.032	0.016	2	0.064	0.25	2	8	0.032	0.002	0.064	8	0.125	0.5
M2/25P1†	A	p083	0.032	0.125	2	0.064	0.5	4	8	0.125	0.004	0.064	16 (R)	1	0.5
M2/25P2†	A	p084	0.032	0.125	2	0.125	0.25	8 (R)	8	0.125	0.008	0.032	16 (R)	2	0.5
M2/25P3†	A	p085	0.032	0.125	2	0.125	0.25	8 (R)	8	0.125	0.004	0.032	16 (R)	1	0.5
M/26P1	A	p039	0.125	0.125	4	0.064	0.25	4	16	0.25	0.004	0.032	8	0.25	1
M/26P2	A	p038	0.032	<0.016	1	0.064	0.25	2	8	0.064	0.002	0.016	8	0.064	0.5
M2/26P1†	A	p100	0.032	0.032	2	1	0.25	4	16	0.125	0.004	0.032	4	0.25	0.5
M2/26P2†	A	p086	0.016	0.064	2	0.125	0.5	4	32 (R)	0.125	0.008	0.032	2	0.125	0.5
M2/26P3†	A (cpb2*)	p093	0.016	0.064	2	0.5	0.5	4	8	0.064	0.004	0.032	4	0.25	0.5
N/01P1	A (cpb2*)	p044	0.125	0.125	2	0.064	0.25	4	16	0.064	0.004	<0.016	8	0.25	1
N/01P2	A (cpb2*)	p045	0.032	0.064	2	0.064	0.5	4	16	0.25	0.004	0.064	8	0.5	0.5
N/01P3	A (cpb2*)	p046	0.032	0.125	4	0.064	0.5	1	8	0.125	0.004	<0.016	8	0.25	0.5
N/12P1	A (*cpe*)	p024	0.064	0.064	2	0.125	0.25	4	16	0.064	0.008	0.032	4	0.5	0.5
N/14P1	A	p019	0.032	0.032	0.25	0.125	0.25	2	8	0.032	0.004	<0.016	4	0.125	0.5
O/04P1	A	p094	0.125	0.064	2	0.25	0.25	4	16	1	0.008	0.032	4	0.125	0.5
O/11P1	A (cpb2*)	p007	0.064	<0.016	0.064	0.064	0.25	4	8	0.125	<0.002	0.016	4	0.125	0.5
O/11P2	A	p009	0.032	0.064	1	0.032	0.25	1	8	0.032	0.004	0.032	8	0.125	0.5
O/11P3	A	p010	0.032	0.064	2	0.064	0.25	2	8	0.064	0.004	0.032	8	0.125	0.5
Q/03P1	A	p073	0.125	0.016	2	0.064	0.25	4	32 (R)	0.064	0.004	<0.016	4	0.125	0.5
Q/03P2	A	p075	0.064	0.032	2	0.125	0.25	8 (R)	8	0.25	0.008	0.032	4	0.125	0.5
Q/03P3	A	p074	0.032	0.125	2	0.125	0.25	4	16	0.125	0.008	0.032	4	0.25	0.5
Q/04P2	A	p059	0.032	0.125	2	0.125	0.25	4	16	0.125	0.004	0.016	4	0.125	0.5
Q2/04P1†	A **(**cpb2*)	p022	<0.016	0.125	2	0.064	0.25	4	16	0.125	0.004	0.016	4	0.25	0.5
Q2/04P3†	A	p098	0.032	0.016	4	0.064	0.25	4	16	0.125	0.004	0.032	2	0.125	0.5
Q/05P1	A (cpb2*)	p048	0.016	<0.016	0.25	0.064	0.5	1	16	0.016	<0.002	<0.016	1	0.125	0.5
Q/05P2	A (cpb2*)	p049	<0.016	0.125	2	0.064	0.5	2	16	0.25	0.004	0.016	4	0.125	0.5

a Isolates whose names only differ in the last number were retrieved from the same animal (e.g. H/05P1, H/05P2 and H/05P3; see Table 3). Daggers (and the number 2 after the clinic's code) indicate isolates that were obtained in the follow-up analysis.

b *cpe*: possession of the gene encoding for enterotoxin. *cpb2*: possession of the gene encoding for β2 toxin, with asterisks indicating atypical forms of the gene (as determined by PCR amplification).

c According to the UPGMA dendrogram shown in Fig. 1 of Álvarez-Pérez et al. [1].

d Minimum inhibitory concentration (MIC). AMC, amoxicillin/clavulanic acid; CLI, clindamycin; ERY, erythromycin; IPM, imipenem; LVX, levofloxacin; LZD, linezolid; MTZ, metronidazole; PEN, benzylpenicillin; RIF, rifampicin; TEC, teicoplanin; TET, tetracycline; TGC, tigecycline; VAN, vancomycin. R: MIC value above the breakpoint for *in vitro* resistance.

**Table 5:** Characteristics of the *Clostridium difficile* isolates obtained in the study.

**Isolate**^a^	**PCR ribotype**^b^	**AFLP genotype**^c^	**Antibiotic susceptibility (MIC, µg/ml)**^d^												
			**AMC**	**CLI**	**ERY**	**IPM**	**LVX**	**LZD**	**MTZ**	**PEN**	**RIF**	**TEC**	**TET**	**TGC**	**VAN**
B/08D1	RT154	d06	1	0.125	0.25	≥32 (R)	≥32 (R)	0.25	0.064	≥32 (R)	<0.002	0.064	0.032	0.016	0.25
B/08D2	RT154	d07	0.25	0.064	0.25	≥32 (R)	≥32 (R)	0.25	0.032	2 (R)	<0.002	0.032	0.032	0.032	0.125
B/08D3	RT154	d05	0.125	0.032	0.064	≥32 (R)	≥32 (R)	0.125	0.032	32 (R)	<0.002	0.064	<0.016	<0.016	0.125
E/03D1	RT?	d13	0.5	≥256 (R)	≥256 (R)	≥32 (R)	≥32 (R)	1	0.032	≥32 (R)	<0.002	0.064	0.5	<0.016	0.125
E/03D2	RT?	d13	0.25	≥256 (R)	≥256 (R)	≥32 (R)	≥32 (R)	0.5	0.064	≥32 (R)	0.004	0.032	1	0.032	0.125
E/03D3	RT?	d14	0.5	≥256 (R)	≥256 (R)	≥32 (R)	≥32 (R)	1	0.125	≥32 (R)	<0.002	0.032	0.5	0.032	0.125
M/14D1	RT106	d15	0.5	0.064	0.125	≥32 (R)	≥32 (R)	0.25	0.064	≥32 (R)	<0.002	0.032	<0.016	<0.016	0.125
M/14D2	RT106	d01	0.25	0.25	1	≥32 (R)	≥32 (R)	0.125	0.064	4 (R)	0.004	0.032	<0.016	<0.016	0.25
M/14D3	RT106	d03	0.5	0.125	0.25	≥32 (R)	≥32 (R)	0.5	0.125	2 (R)	<0.002	0.032	0.032	<0.016	0.125
N/19D1	RT009-like	d08	0.5	0.064	0.25	≥32 (R)	≥32 (R)	0.5	0.032	4 (R)	<0.002	0.064	0.032	<0.016	0.25
N/19D2	RT009-like	d04	0.064	<0.016	0.016	≥32 (R)	≥32 (R)	0.064	<0.016	1	<0.002	0.032	<0.016	<0.016	0.125
N/19D3	RT009-like	d09	0.25	<0.016	0.064	≥32 (R)	≥32 (R)	0.125	<0.016	4 (R)	<0.002	0.032	0.032	<0.016	0.125
N/20D1	RT009-like	d10	0.25	0.064	0.125	≥32 (R)	≥32 (R)	0.25	0.032	≥32 (R)	<0.002	0.032	0.032	<0.016	0.25
N/20D2	RT009-like	d11	1	0.016	0.032	≥32 (R)	≥32 (R)	0.25	0.064	≥32 (R)	<0.002	0.032	<0.016	0.032	0.125
N/20D3	RT009-like	d12	1	0.125	0.25	≥32 (R)	≥32 (R)	0.125	0.064	1	<0.002	0.064	<0.016	<0.016	0.125
Q/03D1	RT106	d01	0.25	0.064	0.064	≥32 (R)	≥32 (R)	0.125	0.032	≥32 (R)	<0.002	0.032	<0.016	<0.016	0.125
Q/03D2	RT106	d02	0.25	0.064	0.25	≥32 (R)	≥32 (R)	0.125	0.032	2 (R)	<0.002	0.064	0.032	0.032	0.25
Q/03D3	RT106	d01	0.25	0.125	0.125	≥32 (R)	≥32 (R)	0.25	0.032	2 (R)	<0.002	0.064	0.064	<0.016	0.25

a Isolates were retrieved from the same animal (B/08D1, B/08D2 and B/08D3; see Table 3).

b Toxin profiles: RT009-like, A-B-CDT- (but with a positive PCR result for *tcdB*); RT106, A+B+CDT-; RT154, A+B+CDT-; RT? (unknown ribotype), A-B-CDT-.

c According to the UPGMA dendrogram shown in Fig. 2 of Álvarez-Pérez et al. [1].

d Minimum inhibitory concentration (MIC). AMC, amoxicillin/clavulanic acid; CLI, clindamycin; ERY, erythromycin; IPM, imipenem; LVX, levofloxacin; LZD, linezolid; MTZ, metronidazole; PEN, benzylpenicillin; RIF, rifampicin; TEC, teicoplanin; TET, tetracycline; TGC, tigecycline; VAN, vancomycin. R: MIC value above the breakpoint for *in vitro* resistance.

## 2 Experimental design, materials and methods

Our survey was performed during one week (from November 24 to December 1, 2015) in a total of 17 primary care veterinary clinics from the Madrid region (Spain). The staff of participating clinics received training for data and sample collection, and email and telephonic support was available throughout the duration of the study. Veterinarians of participating centers were asked to select two swab samples of all feces shed by dogs and cats at their clinic, regardless of the age, origin and clinical condition of the animals, and to send those samples to a central reference laboratory at the Faculty of Veterinary Medicine of Complutense University of Madrid. Additionally, the staff of each participating clinic had to complete a questionnaire of general data about the centre and a second questionnaire for each pair of fecal swabs obtained requesting data on the sample (collection date, consistency of feces and presence of blood) and the animal of origin (species, breed, sex, age, clinical status and episodes of diarrhea and medication(s) within the previous 30 days). An informed consent and agreement to participate in the study was obtained from the owners of each animal before enrolment. Animals were always handled by experienced veterinary practitioners in strict accordance with good animal practice and the Spanish legislation.

The owners of animals yielding a positive culture for *C. difficile* and/or *C. perfringens* were invited to participate in a follow-up survey performed four months after the first study (in March 2016). In this case, fecal swab samples and clinical information of animals was obtained as explained above.

The microbiology procedures used for *C. perfringens* and *C. difficile* isolation from fecal samples, and the methods used for toxin profiling, PCR ribotyping, AFLP subtyping and *in vitro* antimicrobial susceptibility testing of recovered isolates are detailed in our previous publication [1].

## Funding sources

This work was supported by grant AGL2013-46116-R from the Spanish Ministry of Economy and Competitiveness. The funder had no role in study design, data collection and interpretation, or the decision to submit the work for publication.

## References

[1] S. Álvarez-Pérez, J.L. Blanco, C. Harmanus, E.J. Kuijper, M.E. García, Prevalence and characteristics of Clostridium perfringens and Clostridium difficile in dogs and cats attended in divers eveterinary clinics from the Madrid region, Anaerobe (2017) 48, 47-55, 10.1016/j.anaerobe.2017.06.023 10.1016/j.anaerobe.2017.06.023 28687280

